# Effects of Short-Term (20-Day) Alternate-Day Modified Fasting and Time-Restricted Feeding on Fasting Glucose and IGF-1 in Obese Young Women

**DOI:** 10.3390/diseases13120390

**Published:** 2025-12-01

**Authors:** Dian Aristia Rachmayanti, Purwo Sri Rejeki, Raden Argarini, Hermina Novida, Sri Soenarti, Shariff Halim, Chy’as Diuranil Astrid Permataputri, Sheeny Priska Purnomo

**Affiliations:** 1 Master Program of Basic Medical Science, Faculty of Medicine, Universitas Airlangga, Surabaya 60132, East Java, Indonesia; dian.aristia.rachmayanti-2023@fk.unair.ac.id; 2 Faculty of Medicine, Universitas Pembangunan Nasional “Veteran” Jawa Timur, Surabaya 60294, East Java, Indonesia; chyas.diuranil.fk@upnjatim.ac.id; 3 Physiology Division, Department of Medical Physiology and Biochemistry, Faculty of Medicine, Universitas Airlangga, Surabaya 60132, East Java, Indonesia; raden-a@fk.unair.ac.id; 4 Division of Endocrinology, Diabetes and Metabolism, Department of Internal Medicine, Faculty of Medicine, Universitas Airlangga, Surabaya 60131, East Java, Indonesia; hermina-n@fk.unair.ac.id; 5 Geriatric Division, Department of Internal Medicine, Faculty of Medicine, Brawijaya University, Kota Malang 65145, East Java, Indonesia; sri_sunarti.fk@ub.ac.id; 6 Faculty of Health Sciences, University Technology MARA (UiTM) Pulau Pinang, Bertam Campus, Kepala Batas 13200, Pulau Pinang, Malaysia; halimshariff@uitm.edu.my; 7 Faculty of Medicine, Petra Christian University, Surabaya 60236, East Java, Indonesia; sheenypriska@petra.ac.id

**Keywords:** intermittent fasting, TRF, ADMF, IGF-1, FBG, obesity, prediabetes

## Abstract

**Background:** Obesity is a metabolic condition that may impair insulin sensitivity and disrupt glucose homeostasis. Since insulin and glucose affect insulin-like growth factor-1 (IGF-1), disruptions in this axis may elevate the risk of chronic diseases. Intermittent fasting (IF) modulates metabolic parameters, but the impacts on glucose regulation and IGF-1 remain underexplored. This study aimed to assess the short-term effects of two IF types, time-restricted feeding (TRF) and alternate-day modified fasting (ADMF), on fasting blood glucose (FBG) and IGF-1 in obese young women. **Methods:** A quasi-experimental pretest–posttest control group design was conducted over 20 days. The 31 subjects were allocated into ADMF (*n* = 10), TRF (*n* = 11), and Control (*n* = 10). After excluding dropouts and outliers, the final sample consisted of 22 subjects (ADMF = 7, TRF = 8, Control = 7). FBG and IGF-1 serum were measured pre- and post-intervention. **Results:** The FBG post-intervention significantly increased in TRF (*p* = 0.001) and ADMF (*p* = 0.036) groups, but not in Controls. Only the TRF group showed a significant reduction in IGF-1 levels (*p* < 0.001). Nevertheless, the ADMF group exhibited substantial decreases in body weight (*p* = 0.047) and visceral fat (*p* = 0.017). **Conclusions**: A 20-day IF in obese young women induced distinct metabolic effects: TRF lowered IGF-1, ADMF reduced adiposity, and both regimens increased FBG. These findings suggest that early changes in glucose regulation are highly dependent on the specific dietary regimen used. Specifically, TRF predominantly influences endocrine regulation (IGF-1 axis), while ADMF favours adiposity reduction. The concurrent rise in FBG may reflect a transient shift in glucose homeostasis during the early stages of fasting.

## 1. Introduction

Obesity has emerged as a predominant global health issue. In 2022, 890 million people and 160 million adolescents aged 5–19 were affected by obesity. The prevalence is generally greater in women than in males [1,2]. An elevated body mass index (BMI) correlates with a heightened risk and earlier manifestation of numerous non-communicable disorders [3]. Obesity is closely related to the emergence of insulin resistance, a key feature of prediabetes and type 2 diabetes mellitus (T2DM) [4]. It is estimated that up to 70% of individuals with prediabetes will eventually progress to T2DM without appropriate intervention [5]. Notably, recent epidemiological trends indicate a growing burden of prediabetes among young adults, driven by sedentary lifestyles, poor dietary habits, and increased adiposity [6]. In this population, early glycemic impairment is frequently disregarded due to the lack of overt symptoms; nonetheless, it is at this period that preventive therapies are most efficacious [7].

In obesity, an increase in free fatty acids and pro-inflammatory cytokines provokes chronic, low-grade inflammation, which diminishes insulin sensitivity in skeletal muscle, hepatic tissue, and adipose tissue [8]. This progressive deterioration leads to insulin resistance accompanied by hyperglycemia and compensatory hyperinsulinemia, which are the hallmark features of T2DM [9]. In obese individuals, chronic hyperinsulinemia and hypernutrition may promote elevated insulin growth factor-1 (IGF-1) production [10,11]. The IGF-1 serves crucial anabolic functions in growth and metabolism; persistently elevated levels have been associated with increased risk of carcinogenesis and cellular senescence escape [12,13]. Consequently, obesity and diabetes are associated with an increased risk of specific cancer types and other age-related diseases [14,15]. The energy surplus resulting from an imbalance in caloric intake contributes to the development of obesity; therefore, altering dietary habits is essential for its nutritional management [9].

Intermittent fasting (IF) is a dietary approach that involves alternating periods of eating and fasting [16]. This method has demonstrated benefits in the management of obesity and diabetes by reducing body weight, improving glycemic parameters, and decreasing growth factor levels [17,18,19]. The two most widely studied IF methods are time-restricted feeding (TRF), which limits daily calorie consumption to a specific timeframe (6–8 h), and alternate-day modified fasting (ADMF), where individuals alternate between fasting days with around 25% of their caloric intake and unrestricted eating days [20].

Intermittent fasting influences fasting blood glucose (FBG) and IGF-1 levels through multiple mechanisms. The TRF enhances glycemic control by aligning food intake with the circadian rhythm, thereby improving glucose tolerance, insulin sensitivity, and facilitating a transition towards fat oxidation [21,22,23]. Prolonged fasting in ADMF induces a more robust metabolic switch, enhancing glucose and lipid profiles, and facilitating increased fat loss while preserving lean mass [16,24,25,26]. However, the effects of IF on glycemic control are inconsistent, affected by factors including age, health condition, and fasting duration [27,28,29]. Short-term IF influences glucose homeostasis through the interactions of insulin and counter-regulatory hormones [30,31,32,33,34]. Furthermore, the timing of the eating period in IF could impact glucose regulation by interacting with circadian rhythms that govern hormonal secretion and insulin sensitivity [35,36]. Regarding the IGF-1, IF may decrease IGF-1 levels by elevating IGFBP-1, thereby diminishing the bioactivity and bioavailability of IGF-1. Likewise, the downregulation of hepatic GH-signalling components, including GHR and STAT5, during fasting may lead to GH resistance and hinder IGF-1 synthesis [27,37,38,39]. Additionally, reduced insulin availability during fasting may lead to a downregulation of IGF-1 production, which subsequently affects glucose regulation due to the interconnectedness of metabolic pathways. However, the specific mechanisms responsible for the elevation of FBG and the reduction in IGF-1 during TRF and ADMF necessitate additional investigation.

The present study aimed to evaluate the short-term impacts of two IF regimens—TRF and ADMF—on FBG and IGF-1 levels. Given the limited evidence concerning short-term endocrine responses to these fasting protocols, we formulated an exploratory hypothesis proposing that both interventions would elicit alterations in FBG and IGF-1 levels, without implying a particular directional difference between them. This study focused on a metabolically susceptible demographic, notably young women with obesity, as previous IF research primarily involved diverse age groups or individuals with chronic metabolic conditions [31,33,40,41,42]. Investigating this population is crucial, as women demonstrate the highest global prevalence of obesity. Early intervention during this stage may prevent the progression to prediabetes, type 2 diabetes mellitus, and other obesity-related chronic diseases [2,7].

## 2. Materials and Methods

### 2.1. Study Design

**Figure 1:**
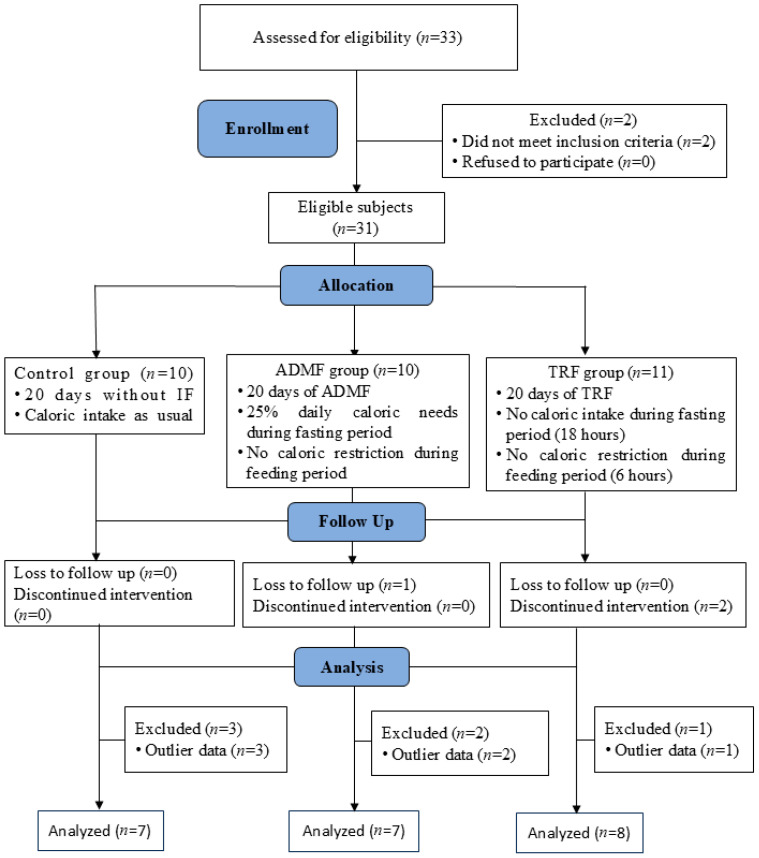
Research workflow. ADMF, Alternate Day Modified Fasting; TRF, Time-Restricted Feeding.

This quasi-experimental study utilized a pretest-posttest control group design. Initially, thirty-three women were evaluated for eligibility. Two subjects were excluded for failing to meet the inclusion criteria, resulting in thirty-one eligible subjects. Thirty-one patients were allocated equally into three groups: Control, TRF, and ADMF, based on their scheduling feasibility. During the 20-day intervention period, participant retention differed among groups. No subjects were lost to follow-up in the Control or TRF groups, but one participant was lost in the ADMF group. No discontinuations were seen in the Control or ADMF groups; however, two subjects withdrew from the TRF group. Subsequent to the intervention, multiple data points were eliminated as outliers, notably three from the Control group, two from the ADMF group, and one from the TRF group. A total of 22 patients were included in the analysis (Control, *n* = 7; TRF, *n* = 8; ADMF, *n* = 7). Figure 1 illustrates the research workflow.

Outcome assessments comprised FBG and serum IGF-1, measured at baseline (pretest) and on day 20 (posttest). Anthropometric and body composition variables were collected concurrently to offer complementary insights into metabolic and physiological changes. Subjects were excluded from the study if they were absent during the screening, pretest, or posttest sessions, or if they developed a severe illness that hindered adherence to at least 80% of the prescribed fasting intervention. Baseline data were collected prior to the initiation of the fasting protocol, and posttest data were gathered one day following the conclusion of the final fasting session. All assessments at both time points were conducted directly by the research team to ensure consistency and measurement accuracy. The Faculty of Medicine Ethics Committee at Airlangga University approved this research (No. 36/EC/KEPK/FKUA/2024). All subjects granted informed consent before data collection commenced.

### 2.2. Subject Characteristics

This research involved female young adults aged 18 to 25 years who fulfilled the inclusion criteria and provided informed consent prior to enrollment. The inclusion criteria were: (1) BMI ≥ 25 kg/m^2^ based on the WHO Asia–Pacific classification; (2) body fat percentage > 30%; (3) nondiabetic status, defined as fasting blood glucose (FBG) < 126 mg/dL; (4) normal blood pressure (systolic < 120 mmHg and diastolic < 80 mmHg); and (5) hemoglobin (Hb) ≥ 12 g/dL.

Subjects were excluded based on the following criteria: (1) diagnosis of chronic conditions including gastritis, diabetes mellitus, hypertension, cardiovascular or endocrine diseases; (2) history of malignancy or smoking; (3) regular intake of medications or supplements that influence glucose metabolism, lipid metabolism, appetite, or hormonal balance; or (4) alcohol consumption, as complete abstinence was required from all subjects.

Dropout criteria were as follows: (1) noncompliance with the researcher’s instructions or absence during screening, pretest, or posttest assessments; (2) onset of a severe illness during the study that hindered continuation of the intervention; or (3) completion of fewer than 80% of the total prescribed fasting days during the intervention period.

### 2.3. Intermittent Fasting Protocol

This research utilized two intermittent fasting regimens: time-restricted feeding (TRF) and alternate-day modified fasting (ADMF). TRF adhered to an 18:6 schedule, wherein subjects fasted for 18 h and ingested their total caloric intake during the subsequent 6-h eating window [43,44]. The TRF group followed an 18-h daily fasting regimen, commencing at 08:00 p.m. and concluding at 02:00 p.m. the subsequent day. Subjects were permitted to consume food ad libitum during the eating period, which spanned from 14:00 to 20:00.

The ADMF group adhered to a 24-h fasting protocol on alternate days, consuming less than 25% of their daily caloric needs, as determined by the Harris-Benedict formula [45,46,47]. A designated catering service supplied meals during fasting days. The composition of the menu, types of food, and methods of preparation were standardized among subjects to ensure consistency in dietary quality. The only variation was in portion sizes, which were tailored to ensure that total caloric intake on fasting days remained within 25% of each participant’s calculated daily energy requirement. During non-fasting days, subjects were allowed unrestricted access to food. Both individuals are permitted to consume plain water during the fasting period. The Control group maintained their standard dietary habits. Prior to the commencement of the study, subjects were provided with standardized instructions to guarantee accurate and consistent data recording. Subjects were directed to record their daily food consumption utilizing an estimated food record approach during the intervention. The research team monitored dietary logs, encompassing portion size, timing, and food type, through daily submissions in individual and group-specific WhatsApp channels to improve adherence tracking.

### 2.4. Outcomes Measurement

#### 2.4.1. Body Composition Assessment

This study assessed anthropometric data, encompassing body weight (kg) and body mass index (BMI), alongside metabolic metrics including body fat percentage (%), visceral fat mass (level), and skeletal muscle mass (%). All measurements were performed using a body composition monitor (Omron HBF-375 Karada Scan Body Fat Composition Analyzer; Omron Healthcare Co., Ltd., Kyoto, Japan) in the morning after an overnight fast. Heart rate and blood pressure were measured using the digital sphygmomanometer (Omron HEM-8712; Omron Healthcare Co., Ltd., Kyoto, Japan).

#### 2.4.2. Blood Sampling and Biochemical Analysis

Biological samples were obtained in the morning, during a comparable menstrual cycle phase, to reduce hormonal variability. Fasting blood glucose (FBG) was assessed after a 10-h overnight fast. Subjects ingested their final meal at 10:00 p.m., and blood samples were obtained the subsequent morning between 8:00 and 10:00 a.m. FBG was assessed from fingertip capillary whole-blood specimens utilizing a plasma-equivalent glucometer (Easy Touch GCU ET322; Zhejiang Easy Touch Medical Instruments Co., Ltd., Wenzhou., China). The device measures within a range of 20–600 mg/dL, demonstrating robust clinical validation with a significant correlation to reference laboratory methods (YSI 2300 STAT analyzer), r^2^ = 0.9571, with 98.3% of readings falling within ±20% of the reference values [48]. The coefficient of variation (CV) for multiple measurements varied between 1.8% and 5.7%, demonstrating strong reproducibility and adherence to the ISO 15197:2013 accuracy standards [48,49].

Blood samples for IGF-1 assay were obtained from the median cubital vein between 08:00 and 10:00 a.m. utilizing a 10 mL Terumo syringe equipped with a 20 G needle. Samples were placed into BD Vacutainer SST II Advance Plus blood collection tubes and centrifuged at 1000 rpm for 5 min at 23 °C to isolate serum from cellular components. All processing processes were finalized within 20 min of collection. Serum concentrations of IGF-1 were evaluated using the Human Insulin-like Growth Factor-1 ELISA kit (catalogue No. E0103 Hu, Bioassay Technology Laboratory, Shanghai Korain Co., Ltd. Shanghai., China). The assay used a sandwich ELISA format, with a detection range of 0.1–40 ng/mL and a sensitivity of 0.058 ng/mL. The intra-assay coefficient of variation (CV) ranged from 5.1% to 7.4%, and the inter-assay CV was less than 8%, demonstrating robust repeatability [50].

### 2.5. Statistical Analysis

The sample size was determined utilizing G*Power version 3.1.9.7. A one-tailed paired-samples t-test was chosen to identify the difference between two dependent means, aligning with the exploratory objective of assessing short-term within-group metabolic responses rather than evaluating between-group superiority. The effect size (dz) was calculated at 0.8, derived from the observed changes in fasting blood glucose and IGF-1 in a comparable intermittent fasting study [51]. The minimum required sample size was 12 subjects, given α = 0.05 and power (1-β) (1-β) = 0.80. In considering a potential attrition rate of approximately 20–25%, the target recruitment was adjusted to 15 subjects, with 5 allocated to each group. This calculation focused on within-group effects, indicating that the study lacked the power to detect between-group differences using ANOVA; thus, these comparisons are interpreted as exploratory.

Statistical analyses were conducted utilizing IBM SPSS Statistics version 27. Data points with standardized Z-scores exceeding +2 or falling below −2 were identified as statistical outliers and omitted from the final analysis. This method is consistent with established statistical practices for relative, distribution-based outlier detection and has been utilized in the IGF-1 study that excluded extreme biomarker values using comparable cutoffs [52,53].

Normality and homogeneity were evaluated with the Shapiro–Wilk and Levene’s tests, respectively. The Brown–Forsythe test was utilized on normally distributed yet non-homogeneous data. For normally distributed and homogenous data, one-way ANOVA with Tukey’s HSD post hoc test was employed for intergroup comparisons, while paired t-tests were utilized for intragroup comparisons. The Kruskal–Wallis test with Dunn’s post hoc test was employed for non-normally distributed data between groups, whereas the Wilcoxon signed-rank test was utilized within groups. Statistical data are expressed as the mean ± standard deviation (SD) for normally distributed variables and as the median with interquartile range (IQR) for non-normally distributed variables. A *p*-value below 0.05 was deemed statistically significant in all analyses.

## 3. Results

### 3.1. Subject Baseline Characteristics

A total of 22 subjects were included in the final analysis (Control *n* = 7, ADMF *n* = 7, TRF *n* = 8). The average age of the subjects was 21.59 ± 1.84 years. At baseline, there were no statistically significant differences among the three groups in terms of age, body weight, BMI, blood pressure, FBG, or IGF-1 serum levels (*p* > 0.05). Table 1 displays the baseline characteristics of the subjects.

Following a 20-day fasting period, the ADMF group demonstrated a significant reduction in body weight, decreasing from 75.24 ± 9.33 kg to 74.29 ± 9.61 kg (*p* = 0.047; *n* = 7; 95% CI: 0.02 to 1.8). Consistent with these findings, visceral fat in the ADMF group also showed a significant reduction from 11 (7–13.5) to 10.5 (7–13) (*p* = 0.017). Nevertheless, no significant changes in body weight or visceral fat were observed in either the Control or the TRF groups. The comparison of body composition data prior to and following the intervention is provided in Table 2.

### 3.2. The Effect of Intermittent Fasting on Fasting Blood Glucose

**Figure 2:**
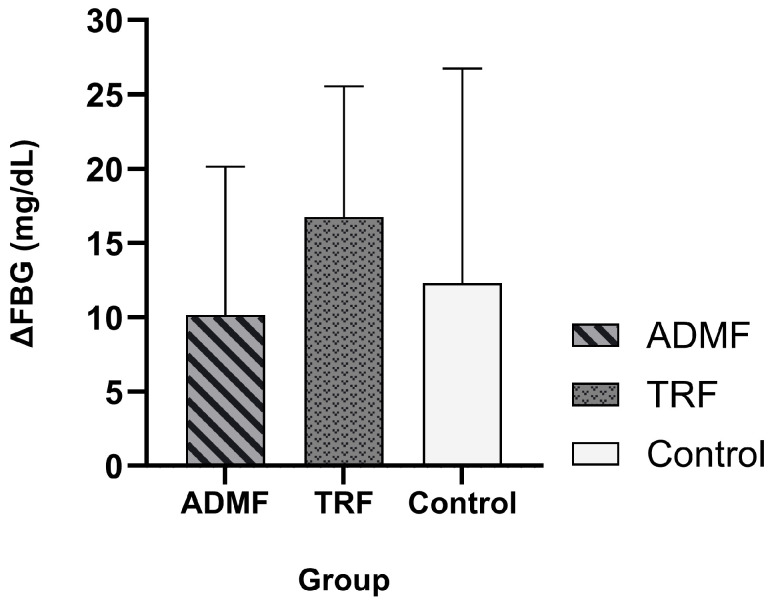
The comparison of changes in FBG levels (ΔFBG) among the three groups was assessed using the One-way ANOVA test. Abbreviation: IGF-1, Insulin-Like Growth Factor-1; ADMF, Alternate-Day Modified Fasting; TRF, Time-Restricted Feeding.

Following 20 days of intervention, both the TRF and ADMF groups demonstrated a statistically significant increase in FBG, whereas the Control group showed no change (*p* = 0.066). FBG levels in the ADMF group increased from 102 ± 10.81 mg/dL to 112.14 ± 6.06 mg/dL (*p* = 0.036; *n* = 7; 95% CI: −19.40 to −0.89). FBG levels in the TRF group rose from 98.42 ± 12.09 mg/dL to 113.71 ± 11.3 mg/dL (*p* = 0.001; *n* = 8; 95% CI: −24.11 to −9.38). However, no statistically significant differences were detected in the comparison of FBG changes between groups (*p* > 0.05). The comparison of FBG levels prior to and following the intervention is displayed in Table 3. The changes in FBG among the three groups are illustrated in Figure 2. Additional statistical results for ΔFBG are provided in Supplementary Table S1.

### 3.3. The Intermittent Fasting Effect on IGF-1 Serum

**Figure 3:**
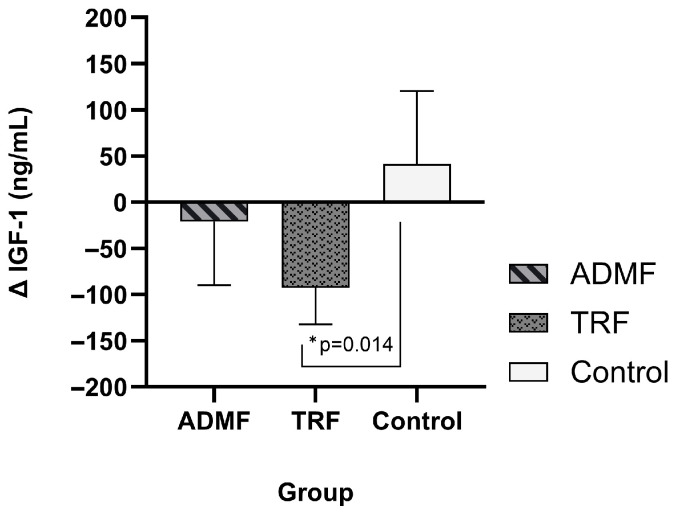
The comparison of changes in IGF-1 serum levels (ΔIGF-1) among the three groups was assessed using the Kruskal–Wallis test, followed by Dunn’s post hoc analysis. Statistical significance (*p* < 0.05) is indicated by an asterisk (*). Abbreviation: IGF-1, Insulin-Like Growth Factor-1; ADMF, Alternate-Day Modified Fasting; TRF, Time-Restricted Feeding.

After 20 days of fasting, the TRF group exhibited a significant decline in serum IGF-1 levels, decreasing from 199.10 (183.32–220.12) ng/mL to 103.94 (83.36–112.27) ng/mL (*p* < 0.001; *n* = 8; 95% CI: 64.08 to 123.97). Conversely, no alterations were observed in the ADMF cohort. In contrast, the Control group exhibited a significant elevation in serum IGF-1 levels, increasing from 120.81 (92.50–164.20) ng/mL to 172.66 (158.49–211.01) ng/mL (*p* = 0.043). The potential of TRF to significantly decrease IGF-1 levels compared to no intervention was demonstrated by the significant difference in IGF-1 change between the TRF and Control groups (*p* < 0.014) in between-group comparisons. The IGF-1 levels prior to and following the intervention are displayed in Table 4. Figure 3 illustrates the changes in IGF-1, detailed comparisons presented in Suplementary Tables S2 and S3.

## 4. Discussion

This research evaluates the short-term impacts of two intermittent fasting regimens—Time-Restricted Feeding (TRF) and Alternate-Day Modified Fasting (ADMF)—on FBG and IGF-1 serum levels in nondiabetic obese young women. The findings revealed a significant increase in FBG following TRF and ADMF interventions, while only TRF produced a significant reduction in IGF-1 levels. Furthermore, ADMF led to greater reductions in body weight and visceral fat, while the TRF group exhibited only a non-significant trend toward reduction in these parameters.

The 20-day duration of this intervention was intentionally designed to examine the effect of short-term ADMF and TRF, rather than their long-term physiological effects. Prior research indicated brief fasting intervention durations; for instance, the study by Hutchison et al. revealed that a 7-day time-restricted feeding intervention in obese males led to a decrease in fasting glucose levels [54]. In addition, the study by Arnason et al. indicated that a 14-day IF protocol in obese adults led to a notable decrease in body weight and a downward trend in fasting insulin levels [55].

The observed increase in FBG contradicts the expected decreases generally associated with caloric restriction or prolonged fasting [31,33,56]. The consequences of intermittent fasting vary among groups, affected by the duration and timing of the fasting period [36]. Multiple studies indicate a temporary increase in blood glucose levels after short-term fasting [30,57]. The paradoxical findings in our study may be due to early changes in glucose regulation that occur during short-term intermittent fasting [43,58].

The implementation of a late eating period, defined as consuming meals exclusively after 4:00 p.m., has been shown to adversely affect postprandial glucose responses [36]. Intermittent fasting protocols that align food intake with the body’s endogenous circadian rhythms enhance metabolic regulation. However, feeding during the biological evening—as seen in late TRF—induces circadian misalignment, leading to unfavourable postprandial outcomes and reduced insulin sensitivity [22,36]. The elevation of blood glucose in this study is contrary to the results of the short-term intermittent fasting trial conducted by Jamshed et al. Eight obese individuals adhering to a four-day early time-restricted feeding regimen (consuming food between 8 a.m. and 2 p.m.) showed significant enhancement in 24-h glucose levels [32]. Circulating hormonal cycles exhibit a circadian pattern, peaking from morning to midday, when insulin sensitivity is at its best; this temporal alignment improves postprandial glucose management when meal consumption occurs earlier in the day [59]. Conversely, postprandial glucose surges may arise following a solitary or short-term late-eating intervention, irrespective of intermittent fasting. Gu et al. established that a late-meal condition elevated postprandial glucose and insulin levels following a single session [60]. Imai et al. reported that three days of late-night supper resulted in postprandial hyperglycemia. Circadian misalignment due to late-evening meals and an extended pre-dinner fast may have exacerbated these responses [60,61].

Fasting transiently impairs first-phase insulin secretion, indicating an adaptive response rather than a pathological one [30]. The initial release of insulin quickly inhibits hepatic glucose production and subsequently enhances peripheral glucose absorption. A decrease in this response is significantly associated with postprandial hyperglycemia, commonly observed in obese individuals and those with impaired glucose tolerance [62]. However, Jørgensen et al. observed that even healthy individuals demonstrated reduced first-phase insulin secretion following 12- and 36-h fasting, thereby supporting the notion of β-cell rest as a physiological adaptation rather than a dysfunction [63]. Likewise, Carlson et al. reported a temporary reduction in first-phase insulin secretion in response to elevated glucose during an eight-week intermittent fasting regimen, which returned to baseline after the intervention ended [30]. In addition to affecting first-phase insulin secretion, Heilbronn et al. conducted a study indicating that three weeks of alternate-day fasting not only influenced first-phase insulin secretion but also resulted in reduced fasting insulin levels in adult men. Nevertheless, this regimen impaired glucose tolerance in women, attributed to decreased insulin sensitivity [64]. Lowered insulin levels and insulin sensitivity may decrease IGF-1 production, which in turn impacts glucose homeostasis, as both hormones regulate glucose through overlapping pathways [11,65].

In addition to alterations in insulin dynamics, fasting induces neuroendocrine adaptations that involve stress hormones, especially cortisol. Cortisol levels in humans increase promptly following the onset of fasting [66]. A meta-analysis indicated a significant correlation between fasting and elevated serum cortisol levels [35]. Cortisol plays a significant role in metabolism during fasting by inhibiting glucose uptake in peripheral tissues, including skeletal muscle and adipose tissue, which results in increased circulating glucose levels. Simultaneously, it promotes glycogenolysis in the liver and muscle, thereby maintaining blood glucose levels [58]. Research involving rats undergoing 24-h fasting indicated an elevation in cortisol secretion amplitude. In contrast, time-restricted feeding (TRF) employing a 16/8 schedule over two weeks and a 22/2 schedule for 20 days resulted in increased cortisol levels and a modification of the acrophase [67,68,69]. Research in humans demonstrated an increase in cortisol levels within the initial 24 h of fasting; likewise, time-restricted feeding for four days also appeared to elevate cortisol levels [32,34,70]. Another study indicated that cortisol levels returned to baseline concentrations approximately one month post-fasting [71].

Notably, an increase in FBG was observed in both the TRF and ADMF groups, despite their differing fasting durations. The 20-day interventions utilized in this study may indicate the initial alterations in glucose regulation associated with short-term intermittent fasting. The findings highlight the need for longer intervention periods to achieve improved glycemic outcomes, as glucose regulation during fasting is influenced by a complex interaction of factors, including body composition, neural inputs, metabolic hormones, and molecular signalling pathways [43,58].

Concerning the influence of short-term IF on IGF-1 in this study, a significant reduction was observed exclusively in the TRF group, but not in the ADMF group. Multiple meta-analyses and systematic reviews indicate that intermittent fasting is linked to decreased serum IGF-1 levels [72,73]. Various biochemical pathways are believed to contribute to this impact, including elevated hepatic IGFBP-1 expression, altered growth hormone receptor (GHR) expression, and compromised GH signalling [37,74,75].

According to a meta-analysis by Rahmani et al., IGFBP-1 levels are raised by intermittent fasting [73]. Despite over 99% of circulating IGFs being associated with IGFBP-3, the initial observable metabolic alteration is frequently an increase in IGFBP-1. IGFBP-1 exhibits a strong affinity for IGF-1. During fasting, a decrease in insulin levels leads to an increase in IGFBP-1 concentrations, which subsequently diminishes the quantity of free, physiologically active IGF-1 [74,76,77]. Human research corroborates this evidence: Caloric restriction for 6 days in healthy people and 14 days in obese women resulted in increased levels of IGFBP-1 and decreased serum IGF-1 [78,79]. A 5:2 diet with a 75% calorie restriction over six months in premenopausal women with obesity dramatically elevated IGFBP-1 and decreased IGF-1 [80]. Nevertheless, the impact of various intermittent fasting protocols, including ADMF and TRF, on IGFBP-1 is still ambiguous.

Fasting markedly reduces circulating insulin levels, leading to a downregulation of hepatic growth hormone receptor (GHR) expression, which is a crucial mediator of GH-induced IGF-1 synthesis in hepatocytes [75,81]. In rodents, a 24-h fasting period resulted in a 50% reduction in hepatic GHR mRNA and a 43% reduction in IGF-1 mRNA, both of which returned to baseline levels following refeeding [39]. Fasting negatively affects GH signalling, especially the JAK-STAT pathway, in which STAT5 is crucial for hepatic IGF-1 production [37,82]. In humans, prolonged fasting (37.5 h) led to a decrease in STAT5 and IGF-1 expression, suggesting impaired GH signalling [37]. However, fasting markedly reduces circulating insulin levels, leading to a downregulation of hepatic growth hormone receptor (GHR) expression, which is a crucial mediator of GH-induced IGF-1 synthesis in hepatocytes [77,83]. In rodents, a 24-h fasting period resulted in a 50% reduction in hepatic GHR mRNA and a 43% reduction in IGF-1 mRNA, both of which returned to baseline levels following refeeding [39]. Fasting negatively affects GH signalling, especially the JAK-STAT pathway, in which STAT5 is crucial for hepatic IGF-1 production [37,84]. In humans, prolonged fasting (37.5 h) led to a decrease in STAT5 and IGF-1 expression, suggesting impaired GH signalling [37]. The impact of ADMF and TRF on GHR expression and GH signalling has not yet been investigated.

The fasting durations implemented—18 h in TRF and 24 h in ADMF—likely contributed to the observed decrease in IGF-1 levels [74]. Insulin decreases approximately six hours postprandially, leading to elevated levels of IGFBP-1 and reduced serum IGF-1 [25,76]. Although TRF does not mandate caloric restriction, the limited daily eating window reliably decreases overall energy intake and insulin levels [83]. Conversely, ADMF decreases calorie consumption by as much as 75% on fasting days, while intake on feeding days is either unchanged or only marginally reduced [84]. Consequently, the ongoing daily reduction in insulin in TRF may lead to a more prolonged increase in IGFBP-1 and a more significant decrease in IGF-1 compared to ADMF.

This study indicates that ADMF resulted in more significant reductions in body weight and visceral fat than TRF, probably due to increased lipolysis during extended fasting periods [25]. Catecholamine levels generally start to increase modestly after 12 h of fasting; however, significant catecholamine-induced lipolysis is observed after roughly 15 h of fasting. The delayed activation may be additionally influenced by reduced catecholamine sensitivity, a condition commonly observed in individuals with obesity [85,86]. This may elucidate the enhanced lipolytic response observed during the 24-h fasting period of ADMF in comparison to TRF [25,87]. The significant decrease in visceral fat indicates a greater lipolytic effect in intra-abdominal adipose tissue compared to subcutaneous fat depots [86]. Moreover, intermittent fasting in this study did not lead to a loss of lean mass, likely due to an initial metabolic shift towards fat and ketone utilization occurring within the first 24 h of fasting, prior to notable protein catabolism [25,36,64]. Thus, the inclusion of 25% of daily caloric intake during fasting periods in the ADMF protocol does not seem to hinder lipolysis or weight loss. This modest intake may enhance adherence to the fasting regimen [84].

To the best of our knowledge, this is the initial study to assess the impacts of various short-term IF protocols on FBG and IGF-1 concentrations in nondiabetic, obese young women. This population is underrepresented in the current literature; yet, studying this group is crucial, as women demonstrate the largest global prevalence of obesity. Timely intervention in obese cases is crucial to avert progression to type 2 diabetes mellitus and other non-communicable disorders [2,7]. This study was designed to capture the early changes in glycemic regulation during short-term intermittent fasting in obese young women [43,58,88].

This research possesses multiple limitations that merit careful consideration. Dietary intake was monitored solely for adherence, without quantification of caloric or macronutrient intake. Physical activity and sleep-circadian patterns were self-reported rather than objectively quantified. Although medication and supplement use were identified through self-reported exclusion criteria, no additional verification was possible, thereby leaving the potential for residual confounding. Although samples were obtained within a comparable morning menstrual cycle window, further hormonal assessments (e.g., insulin, estradiol, progesterone, cortisol) were not feasible, allowing for potential residual hormonal variability. Fasting glucose levels were determined using a capillary glucometer, a technique comparable to those utilized in numerous prior investigations [89,90]. However, in accordance with ISO 15197, these devices may demonstrate a measurement error of ±15–20%, which could partially coincide with the small effect sizes observed. Therefore, slight variations in FBG should be interpreted with caution. Furthermore, the lack of measurements for intermediate metabolic regulators—including insulin, cortisol, and oral glucose tolerance—limits the extent of mechanistic interpretation that can be drawn from the current findings. Future research should employ comprehensive metabolic profiling alongside more rigorous monitoring of behavioural and hormonal confounding factors.

## 5. Conclusions

The 20-day intermittent fasting intervention yielded specific results according to the protocol: Time-Restricted Feeding (TRF) dramatically decreased IGF-1 levels, while Alternate-Day Modified Fasting (ADMF) resulted in reductions in body weight and visceral fat. Both regimens, however, resulted in elevated fasting blood glucose levels, while no alterations were noted in the Control group. These data indicate that early changes in glucose regulation are significantly influenced by the particular dietary regimen employed. These data indicate that even short-term intermittent fasting may elicit an early response in obese young women. Nevertheless, these findings should be regarded with caution owing to various methodological limitations. The observed modifications should be regarded as initial short-term reactions rather than conclusive regulatory effects. Future research necessitates bigger sample sizes, venous-based biochemical tests, extensive metabolic profiling, and stringent control of behavioural and physiological confounders to elucidate underlying mechanisms and enhance causal inference.

## Figures and Tables

**Table 1:** Baseline characteristics of subjects in the ADMF, TRF, and Control groups.

Variable	ADMF (*n* = 7)	TRF (*n* = 8)	Control (*n* = 7)	p-Value
Age, year ^a^	21.14 ± 1.95	22.25 ± 1.98	21.28 ± 1.6	0.664
Systolic BP, mmHg ^a^	111 ± 10.53	119.85 ± 9.45	114.66 ± 8.71	0.585
Diastolic BP, mmHg ^a^	78 ± 3.80	81.42 ± 7.74	82.66 ± 8.14	0.564
hemoglobin, g/dL ^a^	12.62 ± 1.59	12.37 ± 1.33	12.50 ± 1.44	0.947
Weight, kg ^a^	75.24 ± 9.33	76.4 ± 10.43	72.81 ± 12.27	0.809
BMI, kg/m^2 b^	31.4 (27–33.2)	30.7 (27.2–32.07)	28.2 (25.2–30)	0.429
Body Fat, % ^b^	38 (34.6–39.4)	38.05 (34.85–39.2)	36.5 (36.2–38.2)	0.936
Visceral fat rating ^b^	11 (7–13.5)	10.5 (7.62–12.12)	8 (6–10)	0.337
Skeletal muscle, kg ^b^	22.8 (22.4–24)	23 (22.55–25.1)	23.3 (22.1–24.7)	0.697
FBG, mg/dL ^a^	102 ± 10.81	98.62 ± 11.21	101 ± 6.48	0.792
IGF-1, ng/mL ^c^	207.38 ± 104.55	209.4 ± 40.92	131.62 ± 32.9	0.098

Data are reported as mean ± SD for normally distributed variables or as median (IQR1–IQR3) for non-normally distributed variables. ^a^ represents the *p*-value derived from the One-way ANOVA test. ^b^ represents the *p*-value derived from the Kruskal–Wallis test. ^c^ represents the *p*-value derived from the Brown–Forsythe test. Abbreviations: BMI, Body Mass Index; FBG, Fasting Blood Glucose; ADMF, Alternate Day Modified Fasting; TRF, Time-Restricted Feeding; IGF-1, Insulin-Like Growth Factor-1.

**Table 2:** Comparison of body weight, body fat percentage, visceral fat rating, and skeletal muscle mass among the ADMF, TRF, and Control groups.

Variable	Group	Pretest	Posttest	p-Value
Weight, kg	ADMF (*n* = 7) ^b^	75.24 ± 9.33	74.29 ± 9.61	0.047 *
	TRF (*n* = 8) ^b^	76.4 ± 10.43	75.72 ± 10.47	0.222
	Control (*n* = 7) ^b^	72.81 ±12.27	73.4 ± 11.66	0.349
Body Fat, %	ADMF (*n* = 7) ^b^	38 (34.6–39.4)	37.4 (34.4–39.2)	0.370
	TRF (*n* = 8) ^b^	38.05 (34.85–39.2)	38.35 (35.7–38.92)	0.351
	Control (*n* = 7) ^a^	36.5 (36.2–38.2)	35.7 (35.1–39.4)	0.765
Visceral fat rating	ADMF (*n* = 7) ^b^	11 (7–13.5)	10.5 (7–13)	0.017 *
	TRF (*n* = 8) ^a^	10.5 (7.62–12.12)	10.5 (7.45–11.87)	0.739
	Control (*n* = 7) ^a^	8 (6–10)	8 (6–10)	0.785
Skeletal muscle, kg	ADMF (*n* = 7) ^b^	22.8 (22.4–24)	22.7 (20.6–24)	0.498
	TRF (*n* = 8) ^b^	23 (22.55–25.1)	22.95 (22.75–23.32)	0.161
	Control (*n* = 7) ^a^	23.3 (22.1–24.7)	23.5 (22.1–24.3)	0.463

Data are reported as mean ± SD for normally distributed variables or as median (IQR1–IQR3) for non-normally distributed variables. ^a^ denotes the *p*-value derived from the Wilcoxon test. ^b^ denotes the *p*-value derived from the paired *T*-test. An asterisk (*) denotes statistical significance (*p* < 0.05). Abbreviation: ADMF, Alternate Day Modified Fasting; TRF, Time-Restricted Feeding.

**Table 3:** The comparison of FBG levels before and after the intervention in each group.

Variable	Group	Pretest	Posttest	p-Value
FBG (mg/dL)	ADMF (*n* = 7)	102 ± 10.81	112.14 ± 6.06	0.036 *
	TRF (*n* = 8)	98.42 ± 12.09	113.71 ± 11.3	0.001 *
	Control (*n* = 7)	101.5 ± 6.16	108.37 ± 8.37	0.066

Data are reported as mean ± SD. The paired *T*-test was employed to analyze the discrepancy between the pretest and posttest data of FBG. An asterisk (*) denotes the statistical significance (*p* < 0.05). Abbreviation: FBG, Fasting Blood Glucose; ADMF, Alternate Day Modified Fasting; TRF, Time-Restricted Feeding; IGF-1, Insulin-Like Growth Factor-1.

**Table 4:** Comparative analysis of IGF-1 levels between pretest and posttest for each group.

Variable	Group	Pretest	Posttest	p-Value
IGF-1 (ng/mL)	ADMF (*n* = 7) ^b^	205.12 (97.19–312.48)	196.33 (182.45–217.38)	0.995
	TRF (*n* = 8) ^b^	199.10 (183.32–220.12)	103.94 (83.36–112.27)	<0.001 *
	Control (*n* = 7) ^a^	120.81 (92.50–164.20)	172.66 (158.49–211.01)	0.043 *

Data are reported as median (IQR1–IQR3). The difference between pretest and posttest data of the IGF-1 serum was analyzed using the Wilcoxon test (^a^) or paired *T* test (^b^). Statistical significance (*p* < 0.05) is indicated by an asterisk (*). Abbreviation: IGF-1, Insulin-Like Growth Factor-1; ADMF, Alternate-Day Modified Fasting; TRF, Time-Restricted Feeding; IGF-1, Insulin-Like Growth Factor-1.

## Data Availability

Data supporting the reported results can be obtained from the corresponding author upon reasonable request. The data are not openly accessible because of ethical considerations.

## Supplementary Materials

The following supporting information can be downloaded at https://www.mdpi.com/article/10.3390/diseases13120390/s1. Table S1. Comparison of ΔFBG between groups; Table S2. Comparison of ΔIGF-1 between groups; Table S3. Dunn Post-Hoc Test for ΔIGF-1; Table S4. Menu Table for ADMF Group; Figure S1. Food Recording Form; Figure S2. Physical Activity and Sleep Recording Form
