# Supplementary material for: Effects of Short-Term (20-Day) Alternate-Day Modified Fasting and Time-Restricted Feeding on Fasting Glucose and IGF-1 in Obese Young Women
Source: Diseases. 2025 Dec 1;13(12):390. doi: 10.3390/diseases13120390 (PMC12731896; doi:10.3390/diseases13120390)
Supplement: Supplementary file 1 [file diseases-13-00390-s001.zip › diseases-3969519-supplementary.pdf]

Supplementary Data

Effects of Short-Term (20-Day) Alternate-Day Modified Fasting and Time-Restricted Feeding on Fasting Glucose and IGF-1 in Obese Young Women

Tabl.S1 Comparison of ΔFBG between groups

Variable	Group	Mean	Std. Deviation	N	p-Value (ANOVA)
ΔFBG (mg/dL)	Control	12.2857	14.45354	7	
	ADMF	10.1429	10.00714	7	0.518
	TRF	16.7500	8.81152	8	

Statistical analysis using one-way ANOVA indicated that there was no statistically significant difference in the mean changes of fasting blood glucose (ΔFBG) among the three groups (Control, ADMF, and TRF). The analysis showed $p = 0.518$, confirming that the interventions produced **comparable changes** in FBG across groups

Tabl.S2 Comparison of ΔIGF-1 between groups

Variable	Group	Mean Rank	N	p-Value (Kruskal–Wallis)
	Control	16.43	7	
ΔIGF-1 (ng/mL)	ADMF	12.14	7	0.014
	TRF	6.63	8	

Statistical analysis using the Kruskal–Wallis test revealed a statistically significant difference in the median changes of IGF-1 (ΔIGF-1) among the three groups (Control, ADMF, and TRF), with a p-value of 0.014. This indicates that the interventions produced different effects on IGF-1 levels across groups.

Tabl.S3 Dunn Post-Hoc Test for ΔIGF-1

Comparison	Test Statistic	Std. Error	Z	p-value (Dunn Test)
TRF vs ADMF	5.518	3.361	1.642	0.302
TRF vs Control	9.804	3.361	2.917	0.011*
ADMF vs Control	4.286	3.471	1.235	0.651

Post-hoc analysis using Dunn's test revealed that the TRF group showed a significantly greater decrease in ΔIGF-1 compared with the Control group (adjusted $p = 0.011$). No significant differences were observed between TRF vs ADMF or ADMF vs Control.

Tabl.S4 Menu Table

Menu	Sides	Dessert
Oriental Chicken	Brown rice, stir-fried vegetables, green chili tofu	Dragon fruit jelly
Shredded Fish with Basil	Red rice, stir-fried caisim tofu, sautéed green beans with quail tempeh	Gluten-free taro cake
Beef Gyudon	Red rice, pakcoy carrot garlic veggies, hot & sour soup	Lychee jelly
Bangbang Chicken	Rosemary baked baby potatoes, mixed veggies, zucchini chips	Gluten-free carrot cake
Chicken Laksa	Red rice, laksa condiments (vermicelli, bean sprouts), boiled egg	Strawberry jelly
Spicy Chicken & Korean Shrimp	Sweet potato, Asian salad, steamed edamame	Gluten-free banana cake

Menu	Sides	Dessert
Stir-fried Chicken Chili Padi	Red liwet rice, yellow pickles, basil tempeh stir-fry	Grape jelly
Chicken Shashlik	Herb grilled baby potatoes, carrots-beans-cauliflower mix, broccoli fritter	Gluten-free date cake
Chicken Madras	Brown rice, sautéed vegetables, tofu steak	Pineapple jelly
Japanese Hamburg Steak	Red rice, mixed veggies, egg muffin	Gluten-free pumpkin cake
Grilled Fish with Colo-colo Sambal	Yellow rice, long bean & tofu stir-fry, spicy red beans	Guava jelly
Chicken Tsukune	Grilled baby potatoes, salad, maggie gurie tofu	Gluten-free sweet potato cake

Detailed menu provided by the catering service to subjects assigned to the Alternate-Day Modified Fasting (ADMF) group throughout the fasting intervention period.

Fig.S1 Food Recording Form

Food Recording Form

Name :

Group :

Date	Time	Food / Beverage	Food Processing	Portion	Estimated Calori	Additional Note

Standardized food recording form provided to all participants for documenting daily dietary intake throughout the intervention. The form includes fields for date, time, type of food or beverage, food processing method, portion size, estimated calories, and additional notes to support accuracy in dietary assessment.

Fig.S2 Physical Activity and Sleep Recording Form

Physical Activity and Sleep Recording Form

Name :

Group :

Date	Time	Activity	Duration	Calories Burned	Additional Notes

Standardized recording form provided to participants for logging daily physical activities and sleep duration throughout the intervention. The form includes fields for date, time, activity type, activity duration, estimated calories burned, and additional notes to support accurate monitoring of lifestyle behaviors.
